# Twenty‐Three Years of Surveillance in Chinese Avian *Pasteurella multocida* Reveals Declining Antimicrobial Resistance but Increasing Therapeutic Challenges

**DOI:** 10.1155/tbed/2248363

**Published:** 2026-09-15

**Authors:** Nansong Jiang, Tingting Lu, Hongmei Chen, Weiwei Wang, Qizhang Liang, Qiuling Fu, Rongchang Liu, Guanghua Fu, Chunhe Wan, Longfei Cheng, Wensong Jin, Yu Huang

**Affiliations:** ^1^ Research Center for Poultry Diseases, Institute of Animal Husbandry and Veterinary Medicine, Fujian Academy of Agricultural Sciences, Fuzhou 350013, China, faas.cn; ^2^ Fujian Provincial Key Laboratory for Avian Diseases Control and Prevention, Fuzhou 350013, China; ^3^ College of Life Sciences, Fujian Agriculture and Forestry University, Fuzhou 350002, China, fjau.edu.cn

**Keywords:** antimicrobial resistance, avian isolates, biocide tolerance, genotype-phenotype discordance, *Pasteurella multocida*, WGS

## Abstract

*Pasteurella multocida* (Pm) is an important veterinary and zoonotic pathogen that causes significant economic losses in poultry production. However, long‐term surveillance studies integrating antimicrobial resistance (AMR), biocide tolerance, and genomic epidemiology of Pm remain scarce. In this study, we investigated the antimicrobial susceptibility, biocide tolerance, and the phenotypic associations of 136 avian Pm isolates collected from six provinces in China between 2002 and 2024. Whole‐genome sequencing was performed to characterize population structure, identify antimicrobial resistance genes (ARGs), and assess genotype–phenotype concordance. The A:L1:ST129 lineage remained the predominant clone throughout the 23‐year surveillance period, with a high prevalence of AMR‐associated traits observed within this lineage. Although resistance to several commonly used antimicrobial classes declined significantly after 2021, florfenicol resistance continued to increase, suggesting an emerging challenge for the clinical management of pasteurellosis. While the isolates generally exhibited low tolerance to the four representative biocides tested, phenotypic correlations were observed between AMR profiles and biocide tolerance patterns. Furthermore, substantial phenotype–genotype discordance was observed, indicating that the presence of ARGs alone may not be sufficient to accurately predict antimicrobial susceptibility. Overall, this study provides a longitudinal assessment of long‐term AMR trends, biocide tolerance, and genomic epidemiology of avian Pm in China, offering epidemiological evidence for monitoring AMR trends and improving antimicrobial management strategies in poultry production.

## 1. Introduction

*Pasteurella multocida* (Pm), a gram‐negative pathogen comprising at least five capsular serogroups and 16 lipopolysaccharide (LPS) genotypes, infects diverse animal hosts and causes major agricultural losses through diseases including fowl cholera [1]. Pm also poses a zoonotic threat, causing human infections following animal bites or scratches [2]. While antibiotics remain the primary treatment for pasteurellosis, rising antimicrobial resistance (AMR) limits their efficacy. Driven by genomic mutations, antimicrobial resistance genes (ARGs), and metabolic reprogramming, multidrug resistance (MDR) has been a critical challenge across medical and agricultural practices [3]. Given its veterinary and zoonotic impacts, Pm has been listed among the 11 priority pathogens by the European Antimicrobial Resistance Surveillance Networks in Veterinary Medicine [4]. Our previous work identified A:L1:ST129 (capsular serogroup A, LPS genotype L1, and MLST 129) as the dominant clone among avian Pm in China and revealed a high prevalence of resistance genes *tet*(B) and *sul2* [5]. To reduce antimicrobial selection pressure, China implemented a nationwide ban on antibiotic growth promoters in animal feed in 2020 [6], marking a significant transition in antimicrobial management. Nonetheless, the long‐term impact of this change on AMR dynamics in avian Pm remains unclear.

The genotype‐phenotype discordance poses a significant challenge in combating AMR, complicating treatment strategies [7]. For example, the disruption of the *cat* gene via insertion sequences causes *Escherichia coli* and *Klebsiella pneumoniae* susceptible to chloramphenicol despite harboring this gene [8]. Conversely, global transcriptional regulators MarA and SoxS can modulate efflux pump expression and membrane permeability, granting *Escherichia coli* an MDR phenotype without the acquisition of specific ARGs [9, 10]. While this phenomenon has been described in various common pathogens, it remains unexplored in Pm. Furthermore, associations among resistance phenotypes may reflect shared genetic or evolutionary links underlying AMR. Strong phenotypic correlations often indicate the presence of MDR resistance mobile elements or common nonspecific regulatory mechanisms and may provide important clues for identifying emerging resistance mechanisms [11, 12]. When correlations among resistance phenotypes are weak, resistance to different antimicrobials may arise from relatively independent mechanisms, suggesting a lower risk of cross‐resistance and preserving more options for antimicrobial combination therapy [13, 14]. Therefore, monitoring phenotype–phenotype associations and genotype–phenotype concordance in Pm is important for identifying emerging resistance mechanisms and optimizing clinical antimicrobial use.

Beyond antibiotic use, biocide exposure in various environments has emerged as an important driver of AMR as it can lead to both biocide tolerance and cross‐resistance to antibiotics [15]. Biocide tolerance genes are often associated with ARGs on mobile genetic elements, creating strong coselection pressure [16]. Therefore, evaluating biocide tolerance together with AMR is essential for assessing coselection risks, understanding resistance evolution, and improving biosecurity practices in production. To date, few studies have characterized the biocide tolerance profile in Pm, and integrated analyses combining AMR, biocide tolerance, and genomic data remain lacking.

In this study, we characterized the AMR and biocide tolerance profiles of 136 avian Pm isolates collected over a 23‐year period (2002–2024) in China. Our results showed that (i) the A:L1:ST129 lineage remained predominant throughout the surveillance period; (ii) although MDR prevalence and resistance to several antimicrobial classes decreased after 2021, florfenicol resistance continued to increase; (iii) significant associations between AMR profiles and biocide tolerance patterns were observed, suggesting potential links between these phenotypes in Pm populations; and (iv) considerable phenotype–genotype discordance, particularly for aminoglycosides and macrolides, indicated that the detection of ARGs alone may not be sufficient for accurate prediction of antimicrobial susceptibility. These findings provide important evidence for understanding the evolving resistance landscape of avian Pm in China and support the development of more effective treatment strategies, surveillance programs, and biosecurity interventions for poultry production.

## 2. Material and Methods

### 2.1. Bacterial Strains, Culture Conditions, and Reagents

All Pm isolates (*n* = 136) used in this study were previously recovered from diseased poultry in six provinces of China between 2002 and 2024, identified in our laboratory (Supporting Information 1: Table S1 and Supporting Information 2: Figure S1), and preserved by lyophilization. All isolates were collected from clinical cases and epidemiological investigations of poultry diseases. When multiple isolates were identified in the same outbreak, only one representative isolate was included to minimize the overrepresentation of individual outbreaks. *Escherichia coli* ATCC 25922 was used as the quality control strain for antimicrobial susceptibility testing. Unless otherwise stated, all strains were cultured at 37°C for at least 12 h on tryptic soy agar (TSA; BD Difco, Franklin Lakes, NJ, USA) or in tryptic soy broth (TSB; BD Difco, Franklin Lakes, NJ, USA) supplemented with 5% fetal bovine serum (FBS; Gibco, ThermoFisher, Waltham, MA, USA).

All antibiotic agents used in this study, including ceftiofur (CTF), cefotaxime (CTX), amoxicillin‐clavulanic acid (AMC), ampicillin (AMP), gentamicin (GEN), kanamycin (KAN), streptomycin (STR), erythromycin (ERY), tilmicosin (TIL), doxycycline (DOX), tetracycline (TET), tigecycline (TIG), chloramphenicol (CHL), florfenicol (FFC), enrofloxacin (ENR), sulfamethoxazole‐trimethoprim (SXT) were purchased from MedChemExpress (Monmouth Junction, NJ, USA). Additionally, the four evaluated biocides Benzalkonium bromide (BZK), chlorhexidine (CHX), hydrogen peroxide (HP), and glutaraldehyde (GA) were obtained from Aladdin Biochemical Technology Co., Ltd. (Shanghai, China). For clarity and readability, the full names of these agents are used throughout the main text, while their respective abbreviations are exclusively applied to figures and tables.

### 2.2. Antimicrobial Susceptibility Testing

To evaluate the susceptibility profiles of the Pm isolates, the broth microdilution method was conducted according to the Clinical and Laboratory Standards Institute (CLSI) [17], following procedures detailed in our previous work [18]. A total of 16 common antibiotics spanning 7 classes and 4 representative biocides were tested. To ensure rigorous and objective minimum inhibitory concentration (MIC) determination, bacterial growth was quantitatively assessed rather than visual inspection. Briefly, following an 18‐h incubation at 37°C, the optical density at 600 nm (OD600) was measured using a microplate reader (Infinite 200 PRO, Tecan Group Ltd., Männedorf, Switzerland). Wells containing broth served as blank controls to account for background absorbance. The MIC was defined as the lowest concentration resulting in no significant bacterial growth, established by an absorbance threshold of ΔOD600 ≤ 0.05. All tests were performed with three replicates.

The MIC values for the antimicrobial agents were interpreted based on established breakpoints (Supporting Information 1: Table S2). Priority was given to Pm veterinary breakpoints derived from the CLSI VET01S supplement. For agents lacking veterinary‐specific criteria, breakpoints were adopted from the CLSI M45 guidelines. In cases where neither VET01S nor M45 provided definitive thresholds (e.g., for tigecycline), interpretive criteria were adopted from the CLSI M100 guidelines using closely related taxonomic groups (such as *Haemophilus influenzae*) or established quality control strain parameters.

For biocides, the minimum bactericidal concentration (MBC) was additionally determined. Aliquots from the MIC well and the subsequent higher‐concentration wells were plated onto TSA. The MBC was defined as the lowest concentration yielding no viable bacterial colonies after incubation. Given the absence of breakpoints for biocides in both the CLSI [17] and the European Committee on Antimicrobial Susceptibility Testing (EUCAST) guidelines [19], assigning “resistant” or “susceptible” labels is scientifically inappropriate. Instead, *Escherichia coli* ATCC 25,922 was included in biocide assays to provide a reference for comparison.

### 2.3. Whole Genome Sequencing (WGS) and Bioinformatic Analysis

Of the 136 Pm genomes analyzed in this study, 102 were previously sequenced by our group [5, 20], while the remaining 34 were newly sequenced. Genomic DNA was extracted from each isolate utilizing the TIANamp Bacterial Genomic DNA Kit (Tiangen Biotech Co., Ltd., Beijing, China). The purity and concentration of the extracted DNA were quantified using a NanoDrop 2000 spectrophotometer (Thermo Fisher Scientific, Waltham, MA, USA). High‐quality genomic DNA samples were subsequently subjected to WGS on an Illumina NovaSeq 6000 platform (Majorbio Bio‐Pharm Technology Co., Ltd., Shanghai, China) with a 150‐bp paired‐end strategy. The sequencing generated an estimated average coverage depth of 120× per genome. For the raw sequencing reads, quality control and trimming were performed using Fastp v0.22.0 [21]. The reads were subsequently assembled into draft genomes using Unicycler v0.5.1 [22]. The completeness and overall quality of the assembled genomes were assessed utilizing CheckM v2 [23] and QUAST v5.2.0 [24], respectively.

Core‐genome single nucleotide polymorphism (cgSNP) calling and alignment were performed using Snippy v4.6.0 (http://github.com/tseemann/snippy), utilizing a complete reference genome FCF15 (RefSeq Accession Number NZ_CP038871.1). Subsequently, a neighbor‐joining tree was constructed using PopPUNK v2.4.0 [25]. ARGs were identified using the NCBI AMRFinderPlus tool v3.12.8 with default cutoff parameters [26]. MLST was determined by querying the RIRDC scheme within the PubMLST database (https://pubmlst.org/). Additionally, capsule and LPS genotypes were assigned using ABRicate v1.0.1 (http://github.com/tseemann/abricate), employing a previously described method [27], with both the sequence identity and coverage thresholds set to 90%.

### 2.4. Statistical Analyses

All statistical analyses and data visualizations were executed within the R software environment, version 4.2.0 (R Foundation for Statistical Computing, Vienna, Austria). Data manipulation was facilitated by the tidyverse package suite. Cohen’s kappa (κ) coefficient was utilized to evaluate the concordance between AMR genotypes and phenotypes. Concordance was defined as an isolate exhibiting either simultaneous genotypic presence and phenotypic resistance or simultaneous genotypic absence and phenotypic susceptibility. For pairwise comparisons, particularly to quantitatively assess the gap between inhibitory (MIC) and bactericidal (MBC) capacity distributions for biocides, the Wilcoxon rank‐sum test (equivalent to the Mann–Whitney *U* test) was employed. A *p*‐value of <0.05 was considered statistically significant.

## 3. Results

### 3.1. Characterization of the Examined Pm Isolates

A total of 136 avian Pm isolates, collected between 2002 and 2024 from poultry farms across six Chinese provinces (Fujian, Guangdong, Zhejiang, Jiangxi, Beijing, and Hainan), were analyzed in this study (Supporting Information 2: Figure S1). Molecular typing revealed a conserved population structure comprising one identified capsular serogroup, three LPS genotypes, and two known STs (Table S1). The population was dominated by capsular type A (83.1%, 113/136), LPS genotype L1 (94.1%, 128/136), and ST129 (86.8%, 118/136). Overall, the majority of isolates belonged to the A:L1:ST129 clone lineage (*n* = 113), followed by a minor proportion assigned to A:L1:ST342 (*n* = 10), suggesting that the A:L1:ST129 lineage has been successfully maintained in Chinese poultry‐associated Pm populations.

### 3.2. Antimicrobial Susceptibility and Biocidal Tolerance Profiles

**Figure 1:**
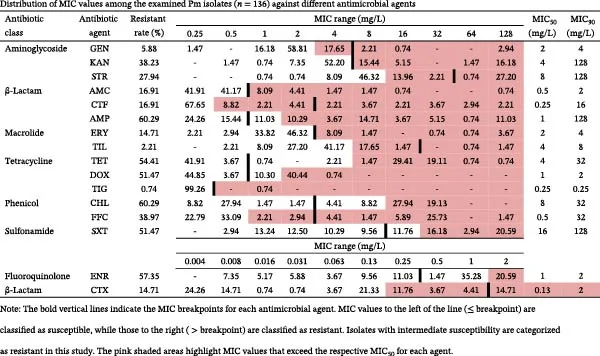
Distribution of MIC values among the examined Pm isolates (*n* = 136) against different antimicrobial agents.

The susceptibility profiles of the 136 Pm isolates against 16 antimicrobial agents revealed highly divergent phenotypic patterns (Figure 1). The highest resistance rates were observed for classical therapeutics ampicillin and chloramphenicol (both 60.29%), alongside enrofloxacin, tetracyclines, and sulfamethoxazole‐trimethoprim, which all demonstrated moderate‐to‐high resistance rates (51.47% to 57.35%). Conversely, striking intraclass differences emerged in β‐lactams. Despite high resistance to ampicillin, the resistance rates of cephalosporins or amoxicillin‐clavulanic acid were below 17%. Both macrolides (erythromycin and tilmicosin) retained potent in vitro activity, with resistance rates remaining low at 14.71% and 2.21%, respectively. Notably, bimodal MIC distributions were observed for sulfamethoxazole‐trimethoprim, florfenicol, and streptomycin. For instance, although the resistance rate for florfenicol was 38.97%, the nonsusceptible strains exhibited high MIC values, driving the MIC_90_ up to 32 mg/L. The divided phenotypes underscore the impact of specific selective pressures, shaping distinct resistant sublineages within a conserved population.

**Figure 2:**
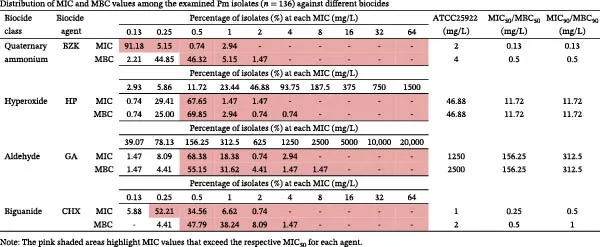
Distribution of MIC and MBC values among the examined Pm isolates (*n* = 136) against different biocides.

To investigate the relationship between AMR and biocide tolerance, the isolates were evaluated against four representative biocides (Figure 2). Overall, the Pm population exhibited low baseline tolerance, with median MICs and MBCs generally remaining lower than those of the reference strain. Benzalkonium bromide and chlorhexidine completely eliminated most isolates at exceedingly low concentrations (MBC_90_ ≤ 1 mg/L). Hydrogen peroxide also demonstrated potent efficacy, effectively inhibiting and killing the majority of isolates at 11.72 mg/L. However, glutaraldehyde displayed a wider variation of susceptibility. While highly effective against the majority, a distinct subpopulation exhibited an extended rightward tail in its MBC distribution (up to 2500 mg/L), indicating greater heterogeneity in biocide tolerance within the population. We further quantitatively compared the MIC and MBC distributions (Supporting Information 2: Figure S2). For benzalkonium bromide, chlorhexidine, and glutaraldehyde, the MBC values were significantly higher than their corresponding MICs, suggesting that higher concentrations are required to achieve bacterial death compared to mere growth inhibition for these biocides. The persistent survival of minor subpopulations at elevated biocide concentrations indicates the presence of highly tolerant subpopulations that warrant further investigation.

### 3.3. Temporal Dynamics of AMR

**Figure 3:**
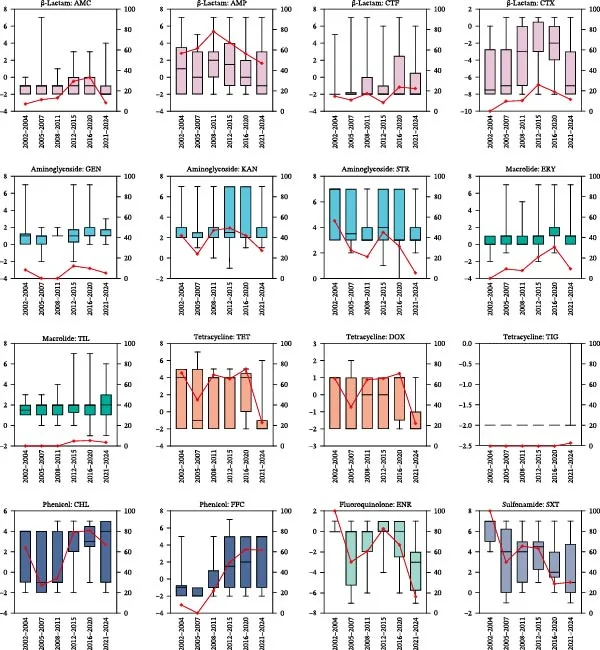
Temporal dynamics of AMR and MIC distributions among Pm isolates (*n* = 136) from 2002 to 2024. The time scale is divided into six intervals. The boxplots represent the distribution density of MICs, plotted on a log_2_‐scale corresponding to the left *y*‐axis. Within each box, the central horizontal line denotes the median, while the upper and lower lines correspond to the 75th and 25th percentiles, respectively. The MIC range extends to the maximum and minimum observed values. The red line graph indicates the continuous fluctuation in the resistance rate (%), referenced to the right *y*‐axis.

To investigate the temporal dynamics of AMR, the MIC or MBC distributions and resistance rates of 16 antimicrobial agents were compared across six sampling periods from 2002 to 2024 (Figure 3 and Supporting Information 1: Table S3). While the temporal dynamics varied across antimicrobial classes, a trend of susceptibility recovery emerged among isolates collected after 2021. This improvement was characterized by reductions in resistance rates and downward shifts in MIC baselines for most agents. Notably, resistance to tetracycline, doxycycline, and enrofloxacin, which remained high prior to 2020, dropped substantially during the 2021–2024 interval. Parallel declines were also observed for combination therapies, including sulfamethoxazole‐trimethoprim and amoxicillin‐clavulanic acid. Accompanying the declining resistance rates, the median MIC values for these agents shifted progressively toward the susceptible range in recent years. For instance, tetracycline MIC distributions shifted toward lower concentrations in recent years, consistent with the observed decline in resistance. Moreover, tigecycline maintained absolute sensitivity throughout the two‐decade span.

Despite these encouraging trends, several critical therapeutics exhibited persistent resistance burdens or alarming deteriorations. The reduction in chloramphenicol resistance was relatively limited. Over the past two decades, the resistance rate of florfenicol climbed continuously, accompanied by a stepwise migration of the Pm population toward higher MIC thresholds. Unlike the recovery seen with tetracyclines, the recent florfenicol MIC distribution remained dispersed, indicating a fragmented population where tolerant mutants coexist with remaining susceptible strains.

We also evaluated the temporal dynamics of biocide tolerance over the same two‐decade period (Supporting Information 2: Figure S3). In contrast to the fluctuations observed in AMR, the bactericidal activities of glutaraldehyde and hydrogen peroxide remained relatively stable throughout the study period. However, the median MBC of benzalkonium bromide increased after 2012. Although the overall median MBC of chlorhexidine remained stable, a subpopulation with higher MBC values emerged in recent years, resulting in an expansion of the upper range of the MBC distributions. Together, these findings suggest that although resistance to several antimicrobial classes has declined in recent years, selection pressure on commonly used antimicrobials and biocides may persist, as reflected by the continued increase in florfenicol resistance and shifts toward higher biocide tolerance in some isolates.

### 3.4. Geographical Differences in Antimicrobial Susceptibility

**Figure 4:**
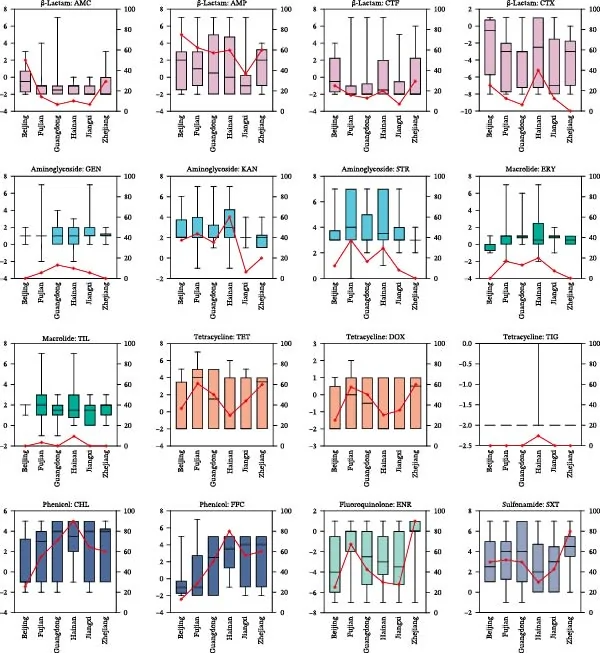
Geographical differences in AMR rates and MIC distributions across six Chinese provinces. The boxplots represent the distribution of log_2_‐transformed MIC values for the tested isolates, plotted against the left *y*‐axis. Within each box, the horizontal line designates the median, and the upper and lower lines denote the 75th and 25th percentiles, respectively. The MIC range extends to the maximum and minimum observed values. The red line graph in each figure traces the corresponding categorical resistance rate (%) for each province, referenced to the right *y*‐axis.

Geographical analysis of the susceptibility profiles revealed marked variation in resistance rates among the Pm isolates from the six provinces (Figure 4 and Supporting Information 1: Table S3). Susceptibility to some antimicrobials, such as tigecycline, was consistently high across all provinces, whereas resistance to several commonly used antimicrobial classes varied considerably by region. For example, isolates from Beijing generally showed lower resistance rates to most antimicrobials but had relatively higher resistance rates and median MICs for β‐lactams. Isolates from Fujian displayed high resistance rates to tetracycline, doxycycline, and enrofloxacin, whereas isolates from Hainan remained relatively susceptible to these three antimicrobials but exhibited high resistance rates to phenicols. Furthermore, Zhejiang isolates showed comparatively high resistance rates to both enrofloxacin and sulfamethoxazole‐trimethoprim.

The MIC distributions also differed among provinces (Figure 4). For some antimicrobial classes with high resistance rates, such as tetracyclines in Fujian, MIC values were distributed across a relatively broad range. In contrast, the enrofloxacin MIC distribution among Zhejiang isolates was more concentrated at higher values. These differences indicate regional variation not only in the proportion of resistant isolates but also in the distribution of MIC values. Geographical differences were also observed in biocide tolerance, although the variation was less pronounced than that observed for antimicrobial susceptibility (Supporting Information 2: Figure S4).

### 3.5. MDR and Phenotypic Coresistance Patterns

**Figure 5:**
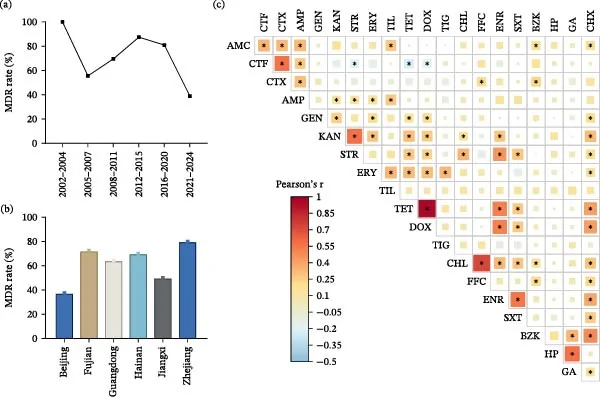
MDR and phenotypic correlation analysis. (a) The line graph reflects the temporal dynamics of the MDR prevalence across six sampling intervals from 2002 to 2024. (b) The bar chart illustrates the geographical difference in MDR rates across the six surveyed provinces. (c) The heatmap visualizes the phenotypic cotolerance network among 16 antimicrobial agents and 4 biocides. Pearson correlation coefficients (*r*) were derived from the log_2_‐transformed MIC values. Positive correlations are indicated by red‐to‐orange squares, whereas negative correlations are denoted by blue squares. The size and color intensity of each square are proportional to the magnitude of the correlation coefficient. Statistically significant correlations (*p* < 0.05) are marked with an asterisk ( ^∗^) within the respective squares.

The prevalence of MDR, defined as resistance to ≥3 antimicrobial classes, varied considerably among Chinese avian Pm isolates over the study period (Figure 5a). MDR prevalence was 100% during the initial 2002–2004 period, fluctuated during the subsequent years, and decreased to below 40% in the most recent 2021–2024 interval. Geographically, the severity of the MDR burden varied among provinces (Figure 5b). Isolates from Beijing exhibited the lowest MDR rate (<40%), whereas Zhejiang and Fujian emerged as the most severely affected provinces, with MDR frequencies approximating 80% and 70%, respectively.

To explore the complex networks underlying MDR phenotype, a Pearson correlation analysis was conducted utilizing the log_2_‐transformed MIC values of all 16 antimicrobials and 4 biocides tested (Figure 5c). In addition to the expected strong intraclass correlations, interclass phenotypic correlations were observed among the tested antibiotics. Notably, strong positive phenotypic correlations were observed among aminoglycosides, macrolides, tetracyclines, and phenicols, with the MIC values of these antimicrobial classes showing significant positive correlations (*p* < 0.05) with one another. Moreover, the correlation matrix unveiled positive correlations between biocide tolerance and antimicrobial MIC values, despite the overall biocide tolerance remaining at a relatively low baseline. Chlorhexidine exhibited widespread, significant positive correlations (*p* < 0.05) with 11 antibiotics. Similarly, benzalkonium bromide demonstrated significant positive correlations specifically with β‐lactams and phenicols.

### 3.6. AMR Phenotype‐Genotype Concordance

**Figure 6:**
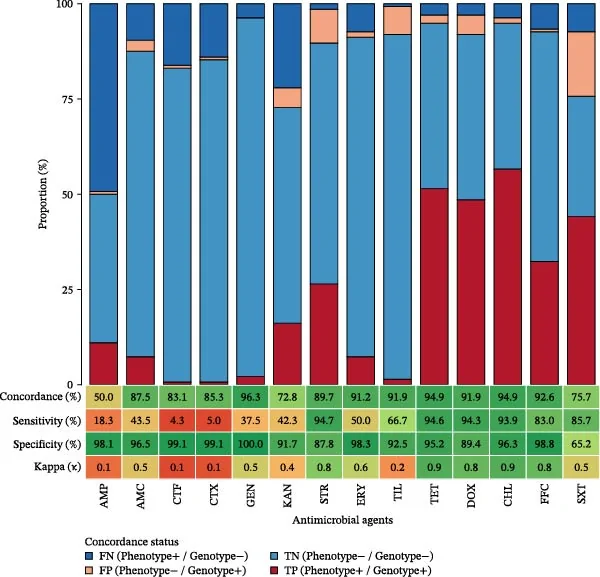
Phenotype‐genotype concordance analysis. The upper stacked bar chart illustrates the proportional distribution of the 136 Pm isolates across four concordance categories: True Positive (TP, red), True Negative (light blue), False Positive (FP, peach), and False Negative (FN, dark blue). The definitions of these categories based on phenotypic and genotypic alignments are detailed in the right‐side legend. Enrofloxacin and tigecycline were excluded from this analysis due to their reliance on mutational resistance and absolute susceptibility, respectively. The aligned heatmap panel below displays the corresponding evaluation metrics for each antimicrobial agent, including overall concordance rate (%), sensitivity (%), specificity (%), and Cohen’s Kappa value (κ). The heatmap cells are color‐coded along a gradient from red (poor concordance) to green (excellent concordance).

To assess the concordance between genomic resistance determinants and antimicrobial phenotypes, we compared WGS‐based ARG detection with phenotypic susceptibility results of the tested antimicrobials (Figure 6). Tigecycline and enrofloxacin were excluded from this matrix. Tigecycline was excluded because nearly all isolates were phenotypically susceptible, providing insufficient phenotypic variation for analysis. Enrofloxacin was excluded because resistance in Pm is primarily associated with chromosomal mutations in quinolone resistance‐determining regions rather than acquired ARGs, which are the focus of the present analysis.

High overall concordance (>89%) and strong Cohen’s kappa coefficients (κ ≥ 0.76) were observed for tetracycline, doxycycline, chloramphenicol, florfenicol, and streptomycin. For these antimicrobials, phenotypic resistance was generally associated with the presence of corresponding acquired resistance genes, including *tet* and *floR* family genes (Supporting Information 2: Figure S5 and Supporting Information 1: Table S4). Sensitivity and specificity were both generally above 85% for these antimicrobial–ARG combinations. Discordance between genotype and phenotype was observed for several other antimicrobials, indicating that ARG detection alone may not fully account for the observed susceptibility profiles. False‐negative results were particularly frequent for β‐lactams. For ampicillin, many isolates showed phenotypic resistance in the absence of detected *bla* family genes, resulting in low genotype–phenotype agreement (κ = 0.10). Similar patterns were observed for the cephalosporins ceftiofur and cefotaxime and for the aminoglycoside kanamycin, for which a relatively high proportion of phenotypically resistant isolates lacked the corresponding resistance determinants identified by WGS.

False‐positive results were also observed for several antimicrobial–ARG combinations. The most pronounced discrepancy was observed for sulfamethoxazole‐trimethoprim, for which a substantial proportion of isolates carried relevant resistance genes, including *sul* and *dfr*, but remained phenotypically susceptible, resulting in a genotypic specificity of 65.2%. Less pronounced genotype–phenotype discordance was also observed for tilmicosin and streptomycin.

### 3.7. Phylogenomic Analysis of the Dominant Clones

**Figure 7:**
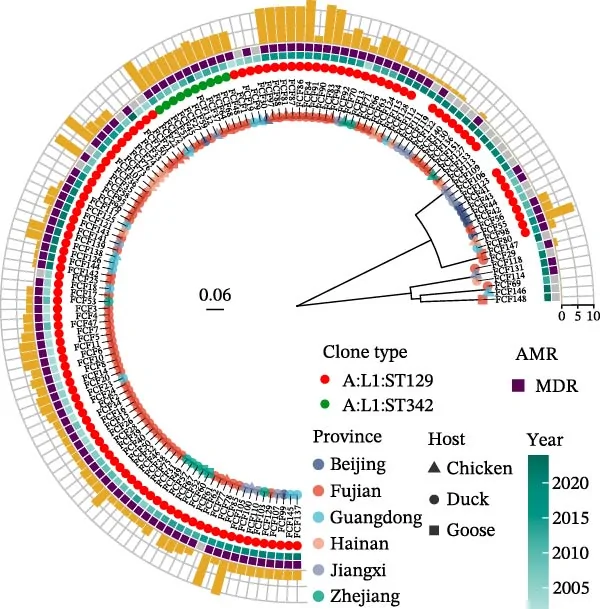
Phylogenetic tree and comprehensive metadata mapping of 136 avian Pm isolates. The neighbor‐joining phylogenetic tree was inferred based on cgSNPs. The scale bar represents 6% core genome difference. The tip nodes are color‐coded and shape‐coded to represent the geographic province of origin and the host species, respectively. The tree is surrounded by four concentric metadata rings (from inner to outer): Ring 1 (red dots) highlights the dominant clonal lineages (A:L1:ST129 and its double‐locus variant ST342). Ring 2 is a color gradient indicating the year of isolation, ranging from 2002 (light teal) to 2024 (dark teal). Ring 3 denotes the MDR status of the isolates (dark purple indicates MDR phenotype). The outermost bar chart (Ring 4, yellow bars) quantifies the total number of acquired ARGs harbored by each corresponding isolate.

To further understand the relationship between the population structure of the dominant Pm clone and AMR, a cgSNP‐based neighbor‐joining tree was constructed encompassing all 136 isolates (Figure 7). The vast majority of the isolates clustered into a single and genetically highly homogeneous clade, characterized by extremely short branch lengths, showing an overwhelmingly clonal population structure. This dominant clade was exclusively composed of the A:L1:ST129 and its double‐locus variant A:L1:ST342 subclones.

Isolates from different provinces and spanning the entire surveillance period intermingled closely within this major clade, indicating the successful, long‐term, and widespread dissemination of A:L1:ST129/ST342 lineage. When AMR metadata were mapped onto the phylogeny, most isolates within the dominant A:L1:ST129/ST342 lineage were classified as MDR and carried a higher number of acquired ARGs. The dense and relatively consistent distribution of acquired ARGs across this lineage was also evident in the phylogenetic tree, whereas the phylogenetically divergent isolates generally carried fewer ARGs and were mostly non‐MDR.

## 4. Discussion

The AMR profiles of Pm vary with host and geographic region [1]. In this study, we examined temporal and geographical variation in AMR and biocide tolerance among 136 Pm isolates collected from six Chinese provinces between 2002 and 2024. The longitudinal design allowed us to assess changes in resistance phenotypes over an extended period while also considering the population structure of the isolates.

Our previous genomic analysis identified A:L1:ST129 as the predominant lineage among avian Pm in China and detected a high prevalence of *tet*(B) and *sul2* [5]. The present study further showed that A:L1:ST129, together with its variant A:L1:ST342, remained prevalent throughout the surveillance period. The recently reported ST471 from Zhejiang is also a variant of ST129 and therefore belongs to Clonal Complex 129 (CC129) [28]. These findings indicate that CC129 has persisted and diversified among the avian Pm population sampled in China. The association between CC129 and AMR was also evident in phylogenomic analysis. Most CC129 isolates were classified as MDR and carried more ARGs, indicating that the dominant lineage has accumulated a greater number of AMR‐associated traits than the more divergent isolates. However, the present data do not establish whether AMR contributed directly to the long‐term persistence of CC129. Other factors, including host adaptation, transmission dynamics, and fitness‐associated traits, may also contribute to the success of this lineage. Further studies are needed to determine the factors underlying the prevalence of CC129.

The AMR profiles revealed that the resistance rates against ampicillin, tetracyclines, sulfamethoxazole‐trimethoprim, chloramphenicol, and enrofloxacin universally exceeded 50%. This high‐burden profile is broadly consistent with several recent surveillance studies of Pm conducted across different hosts and regions, as well as study of *E. coli* from food animals in China [29–31]. Of particular concern is the temporal pattern of florfenicol resistance. While the overall florfenicol resistance rate was 39%, the proportion of resistant isolates increased in the later surveillance period, and the MIC distribution showed evidence of a bimodal pattern, with the upper distribution extending to an MIC_90_ of 32 mg/L. However, resistance to several antimicrobial classes, including tetracyclines and enrofloxacin, decreased among isolates collected after 2021. This observation contrasts with many earlier surveillance reports [32–35], which consistently documented escalating resistance burdens in zoonotic pathogens. Our data align closely with the implementation of China’s national policy restricting the use of antibiotics as growth promoters in animal feed in 2020 [6]. A similar study of *E. coli* from swine likewise found that after the 2017 colistin ban in China, mcr‐1 detection rates significantly decrease, while florfenicol resistance rates increased [31]. The temporal association is consistent with the possibility that reduced veterinary antimicrobial selection pressure contributed to the subsequent decline in resistance. The continued increase in florfenicol resistance is likely attributable to the fact that florfenicol remains one of the few heavily utilized broad‐spectrum antimicrobials approved exclusively for veterinary applications in China. Florfenicol may have become a preferred therapeutic alternative after the restriction of other antimicrobial uses, potentially resulting in greater selective pressure on this specific subpopulation. Consequently, empirical treatments are likely to result in clinical failure against the highly adapted variants.

Our study also highlights geographical disparities in resistance, revealing localized selection pressures. For instance, the exceptional resistance to phenicols observed in Hainan or the severe burden of enrofloxacin resistance in Zhejiang suggest that antimicrobial susceptibility patterns may differ substantially among regions. Even regions with similar resistance rates exhibited different MIC distributions. Highly condensed, high‐concentration MIC populations (e.g., enrofloxacin in Zhejiang) may reflect the predominance of isolates with higher resistance levels in this region. Conversely, regions displaying broader, lower‐level MIC gradients (e.g., tetracyclines in Fujian) indicate an actively changing population, where isolates with different levels of susceptibility may coexist. The regional variation observed here supports the value of incorporating local susceptibility data into antimicrobial treatment decisions in poultry production.

Our study integrated biocide tolerance into the AMR monitoring of Pm. In general, Pm population exhibited low baseline tolerance, remaining susceptible to commonly used farm disinfectants when compared to the reference strain. However, our comparison showed that for benzalkonium bromide, chlorhexidine, and glutaraldehyde, the MBCs were significantly higher than their respective MICs. This gap indicates that concentrations sufficient to inhibit bacterial growth may not be sufficient to achieve complete killing. Positive correlations between several biocide MBCs and antimicrobial MICs were also observed, suggesting that AMR and biocide tolerance may be linked at the phenotypic level. However, correlation alone does not establish cross‐resistance or coselection. Shared mechanisms, such as changes in membrane permeability, efflux activity, or broader stress responses, could potentially contribute to these associations [36, 37], but these mechanisms were not directly investigated in the present study. Therefore, the observed correlations should be considered evidence for a potential relationship rather than direct evidence that biocide exposure selected for AMR. Further experimental studies combining exposure assays, transcriptomic analysis, and targeted genetic characterization are needed to clarify the underlying mechanisms.

The correlation analysis revealed significant positive associations among several antimicrobial classes. Strong interclass correlations were expected because drugs within the same class often share related resistance mechanisms. Positive correlations were also observed between aminoglycosides, macrolides, tetracyclines, and phenicols, indicating that resistance phenotypes across different antimicrobial classes were not distributed independently in the studied population. One possible explanation is the co‐occurrence of the MDR determinants within the same bacterial genetic background. Nevertheless, the correlation analysis itself cannot determine whether the observed phenotypic associations resulted from shared mobile elements, chromosomal mechanisms, regulatory effects, or other population‐level factors.

The phenotype‐genotype concordance analysis further uncovers the complexity of Pm adaptation. While WGS demonstrated excellent predictive reliability for agents like tetracyclines and florfenicol, substantial discordance was observed for several other antimicrobial classes. High false‐negative burden for ampicillin and cephalosporins indicates that phenotypic resistance could not be fully explained by the acquired *bla* genes detected in the present analysis. The underlying mechanisms remain uncertain but may involve undetected resistance determinants, chromosomal alterations, or changes in the expression of resistance‐associated genes, as described for veterinary‐associated Pasteurellaceae [38]. Conversely, the emergence of substantial false‐positive profiles, particularly for sulfamethoxazole‐trimethoprim, highlights the presence of latent resistance reservoirs carrying structurally intact but phenotypically “silent” resistance cassettes (*sul*/*dfr*). This phenomenon likely stems from weak promoters, frameshift mutations, or incomplete in vitro expression, previously corroborated in porcine [39] and bovine Pm [40]. Genotype‐phenotype incongruence may also involve complex regulatory networks, promoter mutations, or plasmid copy number variations. Regardless of the underlying mechanism, silent genes have the potential for rapid reactivation and dissemination under selection pressure [41]. These discrepancies emphasize that relying only on genomic surveillance may underestimate the pathogen’s functional resistance, reaffirming the important role of phenotypic validation.

Despite these findings, several limitations in our study must be acknowledged. First, the uneven distribution of historical isolates across different provinces may partially influence regional prevalence estimations. The collection therefore does not represent a statistically balanced national sampling framework, and regional resistance rates should not be interpreted as population‐level estimates for the entire poultry industry in China. Second, due to the limitation of historical sample collections, detailed metadata regarding the specific farm environments (e.g., historical antibiotic administration, biosecurity practices, and water supply methods) were largely unavailable. The lack of metadata precludes deeper statistical correlation analysis between specific farm management practices and the observed resistance phenotypes [42, 43]. Finally, as our genomic analysis relied primarily on short‐read Illumina sequencing, resolving the complex genomic contexts of specific ARGs proved highly challenging. Building on our findings and acknowledging these limitations, several priorities for future research emerge. Foundational and multidimensional surveillance of AMR and biocide tolerance remains important, particularly within a One Health framework. The genotype‐phenotype discordance identified in this study also warrants further mechanistic investigations, which may help clarify the underlying resistance mechanisms and improve the interpretation of genomic AMR surveillance. Most importantly, the underlying mechanisms enabling the long‐term ecological dominance of the ST129 clone in Chinese poultry require exploration. We must remain vigilant against the potential emergence of A:L1:ST129 as a global “super‐clone,” akin to the notorious Methicillin‐resistant *S. aureus* ST8 [44] or *E. coli* ST131 [45] lineages in human medicine.

In summary, the A:L1:ST129/ST342 lineage remained predominant throughout the surveillance period and was associated with a higher prevalence of MDR and a denser ARG profile. Resistance to several antimicrobial classes declined after 2021, whereas florfenicol resistance continued to increase. The overall low biocide tolerance was accompanied by phenotypic associations with several antimicrobial resistance profiles, although their underlying mechanisms remain unresolved. The observed genotype–phenotype discordance further emphasizes the value of integrating genomic and phenotypic surveillance. Together, these findings support continued regionally informed monitoring of AMR and biocide tolerance in avian Pm.

## Author Contributions

**Nansong Jiang:** data curation, formal analysis, writing – original draft. **Tingting Lu:** data curation, formal analysis, writing – original draft. **Yu Huang:** data curation, project administration, supervision, writing – review and editing. **Hongmei Chen and Longfei Cheng:** formal analysis. **Chunhe Wan, Weiwei Wang, Qizhang Liang, Rongchang Liu, and Qiuling Fu:** investigation. **Wensong Jin:** project administration, supervision, writing – review and editing. **Guanghua Fu:** project administration, supervision.

## Funding

This research was funded by the National Natural Science Foundation of China (Grant 32503051), the Natural Science Foundation of Fujian Province (Grant 2024J01332), the Fujian Academy of Agricultural Sciences Outstanding Talents Program (Grant YCZX202509), the Earmarked Fund for China Agriculture Research System (Grant CARS‐41‐18), the Key Scientific and Technological Project of Fujian Academy of Agricultural Sciences (Grant KJZD202404), and the Fundamental Research funds of Fujian for Public Welfare Research Institutes (Grant 2024R1063).

## Ethics Statement

Ethical approval was not required for this study because all bacterial isolates were recovered from naturally infected dead poultry submitted for routine diagnostic investigations. The present study involved only laboratory analyses of previously collected bacterial isolates and did not include any animal experimentation, intervention, or additional sampling of live animals.

## Conflicts of Interest

The authors declare no conflicts of interest.

## Supporting Information

Additional supporting information can be found online in the Supporting Information section.

## Supporting information


**Supporting Information 1** Table S1: Detailed genomic information for the Pm isolates included in this study. Table S2: Antimicrobial susceptibility breakpoints used in this study and their reference sources. Table S3: Detailed antimicrobial resistance rates of Pm isolates by sampling period and province. Table S4: Distribution of antimicrobial resistance genes among the Pm isolates included in this study.

**Supporting Information 2** Figure S1: Geographic distribution and molecular population structure of the 136 avian Pm isolates. Figure S2: Comparative analysis between the MIC and MBC distributions for the four evaluated biocides. Figure S3: Temporal dynamics of biocide tolerance among Pm isolates from 2002 to 2024. Figure S4: Geographical disparities in biocide tolerance among Pm isolates across six provinces. Figure S5: Prevalence and geographic distribution of ARGs among 136 avian Pm isolates.

## Data Availability

The raw data supporting the conclusions of this article will be made available by the authors upon request. WGS data have been deposited in NCBI under the bioproject PRJNA993376. All gene sequences in this study are available in the NCBI database using the accession numbers in Supporting Information 1: Table S1.
