# Supplementary material for: Twenty‐Three Years of Surveillance in Chinese Avian Pasteurella multocida Reveals Declining Antimicrobial Resistance but Increasing Therapeutic Challenges
Source: Transbound Emerg Dis. 2026 Sep 15;2026:2248363. doi: 10.1155/tbed/2248363 (PMC13575785; doi:10.1155/tbed/2248363)
Supplement: Supplementary file 1 [file TBED-2026-2248363-s002.docx]

**Figure S1.** Geographic distribution and molecular population structure of the 136 avian Pm isolates. The pie charts denote the proportional composition of STs identified within each province. "UT" represents isolates that were untypeable using the standard RIRDC MLST scheme.

**Figure S2.** Comparative analysis between the MIC and MBC distributions for the four evaluated biocides, (a) benzalkonium bromide; (b) chlorhexidine; (c) glutaraldehyde; (d) hydrogen peroxide. Violin plots illustrate the density distribution of the MIC and MBC values, plotted on a log_2_-transformed scale to facilitate visual comparison. Within each violin, the solid red line represents the median, while the black dotted lines denote the upper and lower quartiles. The horizontal black dashed line at y=0 corresponds to the baseline reference concentration of 1 mg/L. Statistical significance regarding the shift between the MIC and MBC population distributions was determined using the Mann-Whitney U test. p < 0.05 was considered statistically significant (** p < 0.01; **** p < 0.0001; ns, not significant).

**Figure S3.** Temporal dynamics of biocide tolerance among Pm isolates from 2002 to 2024. The violin plots illustrate the density distributions of log2-transformed minimum bactericidal MBCs for four evaluated biocides: (a) benzalkonium bromide, (b) chlorhexidine, (c) glutaraldehyde, and (d) hydrogen peroxide, stratified across six chronological intervals. Within each violin, the solid black horizontal line represents the median, while the dotted lines indicate the upper and lower quartiles (interquartile range).

**Figure S4.** Geographical disparities in biocide tolerance among Pm isolates across six provinces. The violin plots depict the distribution of log2-transformed MBCs for (a) benzalkonium bromide, (b) chlorhexidine, (c) glutaraldehyde, and (d) hydrogen peroxide. Within each violin, the solid black horizontal line represents the median, while the dotted lines indicate the upper and lower quartiles (interquartile range).

**Figure S5.** Prevalence and geographic distribution of ARGs among 136 avian Pm isolates. (a) The bar chart displays the overall detection frequencies of the 25 identified acquired ARGs and the biocide tolerance gene *qacL* across the Pm population. (b) The heatmap illustrates the carriage counts of each resistance determinant stratified by the six surveyed provinces. The color intensity within each cell corresponds to the number of isolates harboring the respective gene, ranging from white (zero isolates) to dark blue (highest abundance, >40 isolates), as indicated by the right-side color scale.
